# Qualitative and quantitative analysis of the callosal projections to prefrontal, frontal motor, and parietal areas in the macaque monkey

**DOI:** 10.1007/s00429-025-03060-x

**Published:** 2025-12-17

**Authors:** Marianna Rizzo, Giuseppe Luppino, Elena Borra

**Affiliations:** Dipartimento di Medicina e Chirurgia, Unità di Neuroscienze, Università di Parma, via Volturno 39, Parma, 43125 Parma Italy

**Keywords:** Corpus callosum, Interhemispheric connectivity, Homotopic connections, Heterotopic connections, Bimanual coordination, Neural tracers

## Abstract

In the macaque brain most cortical areas are connected through the corpus callosum to the same or different areas of the contralateral hemisphere (homotopic and heterotopic callosal connections). Many studies have described the callosal connectivity of several cortical areas, but the proportion of callosal vs. ipsilateral afferences and, except for frontal motor areas, of homotopic vs. heterotopic afferences is still unknown. We have analyzed qualitatively and quantitatively the distribution of callosal projecting neurons (CPNs) after neural tracer injections in prefrontal, frontal motor and opercular, and parietal areas (36 tracer injections in 20 macaques). The percentage of CPNs with respect to the total number of labeled neurons (ipsi + contra), with few exceptions, was at least 5% and though quite variable tended to be higher for prefrontal (~ 17%) and premotor (~ 14%), and lower for parietal (~ 9%) areas. For most areas, heterotopic afferences were richer than homotopic ones and for some (e.g., F6 and F3) the areal distribution of CPNs was almost similar to that of the ipsilateral labeling. Furthermore, in areas 24, F6, and F3 the amount of CPNs tended to be relatively high. Finally, the laminar distribution of CPNs could differ from that of the ipsilateral labeling and varied according to the projecting and/or the target area. The present data provide a general framework, though still not complete, of the interhemispheric connectivity in the primate brain and could be useful for a better understanding of the interhemispheric interactions in bimanual coordination, sensorimotor integration, and cognitive functions.

**Supplementary Information:**

The online version contains supplementary material available at 10.1007/s00429-025-03060-x.

## Introduction

In the mammal brain, the corpus callosum is the largest commissure connecting the two cerebral hemispheres and is considered essential for bilateral sensory and motor integration and higher cognitive functions.

Through the corpus callosum almost any cortical area can establish connections with the corresponding one of the contralateral hemisphere (homotopic callosal projections), with other areas of the contralateral hemisphere (heterotopic callosal projections), and with the contralateral striatum (crossed corticostriatal projections). The distribution pattern of the heterotopic callosal connectivity appears to only partially reproduce the pattern of the ipsilateral one.

Early studies based on partial or complete section of the corpus callosum first described the overall pattern of callosal connectivity in non-human primates (e.g., Pandya et al. 1971; Karol and Pandya 1971). Several subsequent studies based on neural tracers injections in the non-human primate brain have described the callosal connectivity of the prefrontal (Schwartz and Goldmann-Rakic 1982, 1984; Barbas and Pandya 1984), frontal motor (Gould et al. 1986; Rouiller et al. 1994; Liu et al. 2002; Marconi et al. 2003; Boussaoud et al. 2005; Lanz et al. 2017; Dancause et al. 2007; Jenny 1979; Muakkassa and Strick 1979; Leichnetz 1986; Jones et al. 1979), somatosensory (Killackey et al. 1983; Manzoni et al. 1984, 1986), posterior parietal (Caminiti and Sbriccoli 1985; Seltzer and Pandya 1983; Andersen et al. 1985; Neal 1990), temporal (Cipolloni and Pandya 1985), and extrastriate visual (Van Essen et al. 1982) areas.

However, none of these studies has provided quantitative data on the contribution of the callosal afferences to the overall cortical connectivity of the various studied areas. Furthermore, except for some studies focused on frontal motor areas (Rouiller et al. 1994; Liu et al. 2002; Marconi et al. 2003; Boussaoud et al. 2005; Lanz et al. 2017), no quantitative data have been provided on the relative contribution of the homotopic vs. heterotopic callosal afferences and the relative contribution of the various areas to the heterotopic callosal afferences to a specific cortical area. Data on the relative contribution of homotopic vs. heterotopic connections to different brain regions have been provided by Szczupak et al. (2023), based however on diffusion-weighted imaging. Specifically, this study suggested that in primates about 75% of the callosal connectivity is heterotopic. This relatively unexpected finding needs to be examined in more detail, preferably based on neural tracers data. In this regard, it is worth noting that the crossed corticostriatal projections can vary in their strength based on their cortical origin and striatal termination (Borra et al. 2022).

Accordingly, the aim of this study was to describe qualitatively and quantitatively the callosal connectivity of the macaque brain based on retrograde neural tracers injections in several prefrontal, frontal motor, frontal opercular, and parietal areas.

Preliminary data were previously published in the abstract form (Rizzo et al. 2024).

## Methods

### Subjects, surgical procedures, and injection sites

In the present study, the cortical callosal connectivity has been analyzed based on the results from retrograde tracer injections in 20 macaque monkeys (5 *Macaca fascicularis*, 5 *Macaca nemestrina*, and 10 *Macaca mulatta*), in prefrontal, frontal motor, frontal opercular, and parietal areas, listed in Table 1. As most of these cases have been already used in previous studies focused on the ipsilateral connectivity (see for references Table 1), in this section the description of surgical procedures and histological processing will be partially reused.

Animal handling as well as surgical and experimental procedures complied with the European (directives 86/609/EEC, 2003/65/CE, and 2010/63/EU) and Italian (D.L. 116/92 and 26/2014) laws in force on the humane care and use of laboratory animals. All procedures were approved by the Veterinarian Animal Care and Use Committee of the University of Parma and authorized by the Italian Ministry of Health.

Under general anesthesia and aseptic conditions, each animal was placed in a stereotaxic apparatus and an incision was made in the scalp. The skull was trephined to remove the bone, and the dura was opened to expose a small cortical region. After the neural tracer injections, the dura flap was sutured, the bone flap replaced, and the superficial tissues sutured in layers. During surgery, hydration was maintained with saline, and heart rate, blood pressure, ventilation, and body temperature were continuously monitored. Upon recovery from anesthesia, the animals were returned to their home cages and closely observed. Dexamethasone (0.5 mg/kg, i.m.) and prophylactic broad-spectrum antibiotics (e.g., Ceftriaxone 80 mg/kg, i.m.) were administered pre- and postoperatively, as were analgesics (e.g., Ketoprofen 3 mg/kg, i.m.).

### Tracer injections and histological procedures

**Fig. 1:**
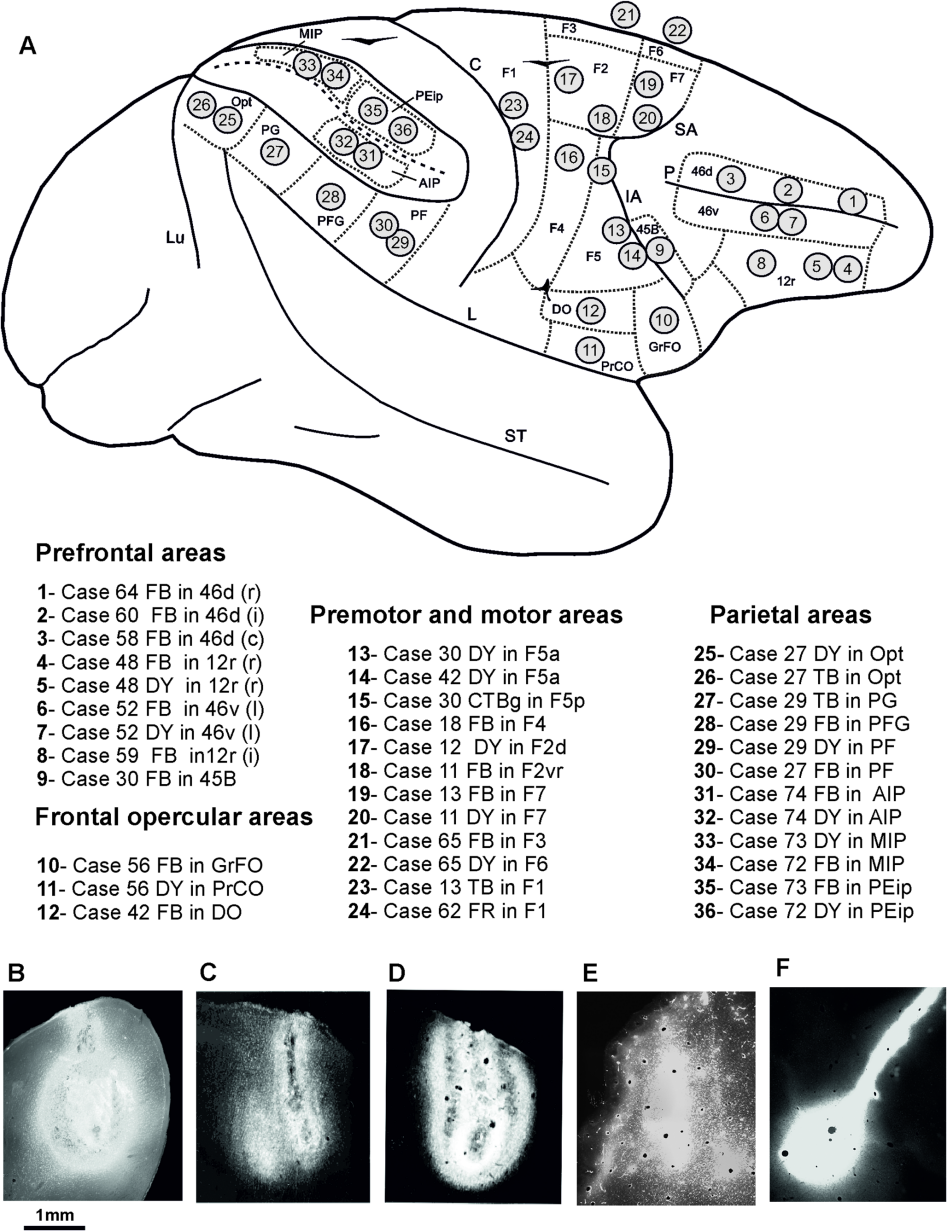
A: Summary view of the location of the cortical injection sites mapped onto a drawing of a template hemisphere. The intraparietal sulcus is “opened” to show the location of the injection sites in the two banks. B–F Photomicrographs showing typical injection sites visualized with fluorescent microscopy; B FB injection site in 46d in Case 60; C: DY injection site in F2d in Case 12; D TB injection site in area F1 in Case 13; E FR injection site in F1 in Case 62; F CTBg injection site in F5p in Case 30. C, central sulcus; c, caudal; i, intermediate; IA, inferior arcuate sulcus; IP, intraparietal sulcus; L, lateral fissure; Lu, lunate sulcus; P, principal sulcus; r, rostral; SA, superior arcuate sulcus; ST, superior temporal sulcus

Once the appropriate injection site was chosen, the neural tracers Fast Blue (FB, 3% in distilled water, Dr Illing Plastics GmbH, Breuberg, Germany), Diamidino Yellow (DY, 2% in 0.2 M phosphate buffer at pH 7.2, Dr Illing Plastics), True Blue (TB, 5% in distilled water, EMS-POLYLOY GmbH, Gross-Umstadt, Germany), Dextran conjugated with tetramethylrhodamine (Fluoro-Ruby, FR, 10% 0.1 M phosphate buffer, pH 7.4; Invitrogen), Cholera Toxin B subunit conjugated with Alexa 488 (CTB-green, CTBg, 1% in 0.01 m phosphate-buffered saline, pH 7.4, Invitrogen, Thermo Fisher Scientific), were slowly pressure-injected through a glass micropipette (tip diameter: 50–100 μm) attached to a 1-, 5-, or 10-µL Hamilton microsyringe, positioned with a stereotaxic holder. Table 1; Fig. 1 summarize the locations of the injections, the injected tracers, and the amounts injected.

After appropriate survival periods following the injections (15–28 days), each animal was deeply anesthetized with an overdose of sodium thiopental (200 mg/kg) and perfused through the left cardiac ventricle consecutively with saline (about 2 L in 10 min), 3.5% formaldehyde (5 L in 30 min), and 5% glycerol (3 L in 20 min), all prepared in 0.1 M phosphate buffer, pH 7.4. Each brain was then blocked coronally on a stereotaxic apparatus, removed from the skull, photographed, and placed in 10% buffered glycerol for 3 days and 20% buffered glycerol for 4 days. Finally, each brain was cut frozen into coronal sections of 60-µm or 50-µm (Cases 62 and 64) thickness.

In all cases in which fluorescent neural tracers were injected (FB, DY, TB, CTBg), sections spaced 300 μm apart (that is one section in each repeating series of 6 in Cases 62 and 64, or of 5 in the other cases) were mounted, air-dried, and quickly coverslipped for fluorescence microscopy. In Case 62, one section in each repeating series of 6 was processed for the visualization of FR. Specifically, in all sections of this series endogenous peroxidase activity was eliminated by incubation in a solution of 0.6% hydrogen peroxide and 80% methanol for 15 min at room temperature. Subsequently the sections were incubated for 72 h at 4 °C in a primary antibody solution of rabbit anti-FR, in 0.5% Triton, 5% normal goat serum (Vector Laboratories) in PBS. The sections were then incubated for 1 h in biotinylated secondary antibody (1:200, Vector Laboratories) in 0.3% Triton, 5% normal goat serum in PBS. Finally, FR labeling was visualized using the Vectastain ABC kit and the Vector SG peroxidase substrate kit (SK-4700, Vector Laboratories) as a chromogen.

In all cases, sections spaced 300 μm apart were stained with the Nissl method (0.1% thionin in 0.1 M acetate buffer, pH 3.7).

**Table 1:** Animals used, area of injection sites, and type and amount of injected tracers

Case	Species (Macaca)	Sex	Age (years)	Hemisphere	Area	Tracer	Amount
Prefrontal areas							
30	*Nemestrina*	M	20	R	45B^a^	FB 3%	0.2 µl
48	*Mulatta*	M	9	R	12r rostral^b^	FB 3%	0.2 µl
					12r rostral^b^	DY 2%	0.2 µl
52	*Mulatta*	M	11	R	46v int^c^	FB 3%	0.2 µl
					46v int^c^	DY 2%	0.2 µl
58	*Fascicularis*	F	9	R	46d caudal^d^	FB 3%	0.2 µl
59	*Mulatta*	M	8	R	12r int	FB 3%	0.2 µl
60	*Fascicularis*	M	3.5	L	46d int^d^	FB 3%	0.3 µl
64	*Fascicularis*	F	4	R	46d rostral^d^	FB 3%	0.2 µl
Frontal motor and opercular areas							
11	*Nemestrina*	F	11	R	F7^e^	DY 2%	2 × 0.2 µl
					F2^e^	FB 3%	0.2 µl
12	*Nemestrina*	M	11	L	F2^e^	DY 2%	0.2 + 1.5 µl
13	*Fascicularis*	F	12	L	F7^e^	FB 3%	0.2 µl
					F1^e^	TB 5%	0.2 µl
18	*Nemestrina*	M	14	L	F4	FB 3%	0.2 µl
30	*Nemestrina*	M	20	R	F5a^f^	DY 2%	0.2 µl
					F5p	CTB 1%	2 × 1 µl
42	*Mulatta*	M	7.5	R	F5a^f^	DY 2%	0.2 µl
					DO^g^	FB 3%	0.2 µl
56	*Mulatta*	M	4.5	R	GrFO^g^	FB 3%	0.2 µl
					PrCO^g^	DY 2%	0.2 µl
62	*Mulatta*	F	4	L	F1	FR 10%	2 × 1 µl
65	*Mulatta*	M	12.5	R	F3	FB 3%	0.2 µl
					F6	DY 2%	0.2 µl
Parietal areas							
27	*Nemestrina*	F	9	R	PF^h^	FB 3%	0.2 µl
					Opt^h^	DY 2%	0.2 µl
					Opt^h^	TB 5%	0.2 µl
29	*Fascicularis*	F	7	R	PF^h^	DY 2%	0.2 µl
					PFG^h^	FB 3%	0.2 µl
					PG^h^	TB 5%	0.2 µl
72	*Mulatta*	M	9	R	PEip ^i^	DY 2%	2 × 0.15 µl
					MIP ^i^	FB 3%	2 × 0.15 µl
73	*Mulatta*	M	9	L	PEip ^i^	FB 3%	0.3 µl
					MIP ^i^	DY 2%	0.3 µl
74	*Mulatta*	M	15.5	L	AIP ^j^	FB 3%	0.2 µl
					AIP ^j^	DY 2%	0.2 µl

Ipsilateral cortical connections described in: ^a^Gerbella et al. (2010), ^b^Borra et al. (2011), _c_Gerbella et al. (2013), ^d^Borra et al. (2019), ^e^Matelli et al. (1998), ^f^Gerbella et al. (2011), ^g^Gerbella et al. (2016), ^h^Rozzi et al. (2006), ^i^Caminiti et al. (2021), ^j^Lanzilotto et al. (2019)

### Data analysis

All the injection sites used in this study were completely confined to the cortical grey matter. For the areal attribution of the injection sites and of the labeled neurons, the cortex was subdivided according to architectonic or connectional criteria described in detail in Caminiti et al. (2017). The cortical distribution of retrograde labeling in the ipsilateral and contralateral hemispheres was plotted in sections every 600 μm together with the outer and inner cortical borders, using a computer-based charting system.

### Quantitative analysis

In all cases, the number of labeled cells plotted in the ipsilateral hemisphere outside the injected cortical area and in the contralateral hemisphere was counted and the contribution of the callosal projections was expressed in terms of percentage of callosal projecting neurons (CPNs) with respect to the total number of labeled neurons (ipsi + contra). The correlation between the percentage of CPNs and the total number of labeled neurons (ipsi + contra) was evaluated for all injection cases using Pearson’s correlation. Furthermore, the amount of CPNs relative to the total amount of labeled neurons observed after tracer injections in the parietal, frontal motor, and prefrontal areas was compared using a negative binomial regression model, as the data distribution showed overdispersion. For each comparison between groups, we obtained the Rate Ratios and the p-values adjusted for multiple comparisons using the Benjamini and Hochberg procedure to control the false discovery rate. Then, the contribution of the homotopic projections was calculated in terms of percentage of CPNs in the area homotopic to the injected one with respect to the overall number of CPNs. The correlation between the percentage of CPNs and that of homotopic CPNs was evaluated for all injection cases using Pearson’s correlation. Furthermore, in each hemisphere, the percentage distribution of the labeling in the various cortical areas was calculated, excluding the injected area in the ipsilateral hemisphere and the homotopic area in the contralateral one. Finally, in each case, for the cortical areas in which the ipsilateral labeling was > 1%, the number of CPNs was divided by the number of labeled neurons observed in the corresponding ipsilateral area, obtaining a laterality index. This index could range from zero (all cells in the ipsilateral area) to one (equal number of cells in the ipsilateral and contralateral area), or more (more cells in the contralateral area).

### Laminar distribution of the labeling

To obtain information about the organization of the laminar patterns of the observed connections, the labeling attributed to a given area and reliably observed across different sections and cases was analyzed in sections at every 300 μm in terms of percentage of retrogradely labeled neurons located in the superficial (II-III) versus deep (V-VI) layers (s/d ratio). The data were then interpreted according to the model proposed by Felleman and Van Essen (1991) in which the projection from the labeled area to the injected area is *feedforward* when labeled neurons are located mainly in the superficial layers, *feedback* when labeled neurons are located mainly in the deep layers, and *lateral* when labeled neurons are more equally distributed. Laminar distribution patterns of CPNs were then compared with those observed in the ipsilateral areas as described in the studies listed in Table 1.

## Results

**Fig. 2:**
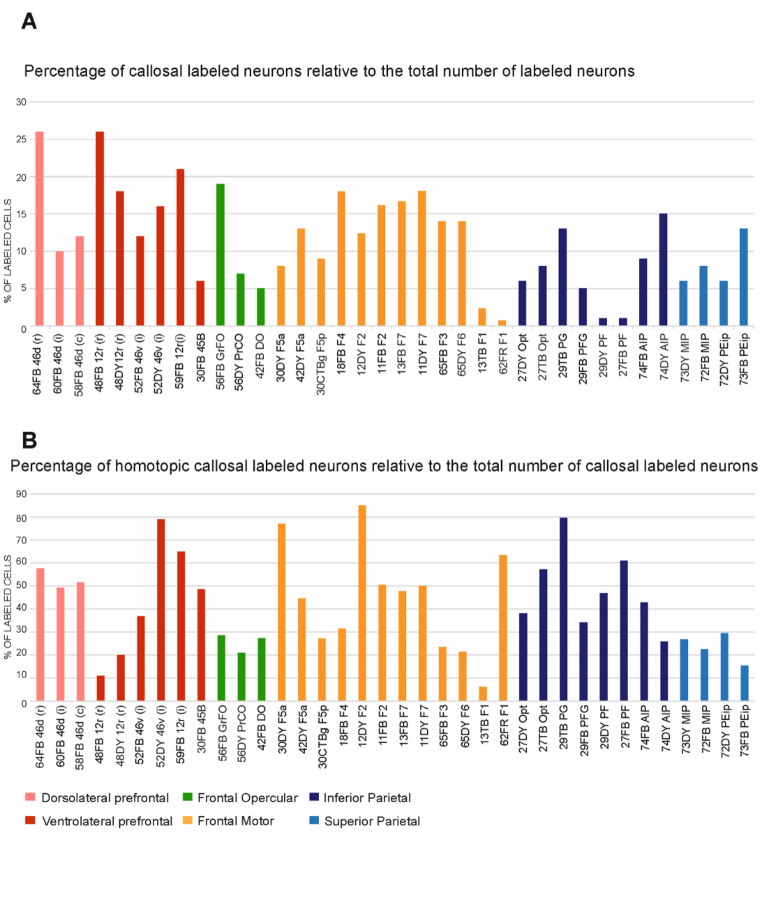
A: Percentage of callosal labeled neurons relative to the total number of labeled neurons (ipsi + contra = 100%) in all studied cases. **B** Percentage of labeled CPNs observed in the homotopic area relative to the total number of CPNs (contra = 100%) in all studied cases. Abbreviations as in Fig. 1

Figure 2A shows the percentage of labeled CPNs with respect to the total number of labeled neurons (ipsi + contra) in all the cases under study. Except for the cases of tracer injections in F1 (Cases 13 TB and 62 FR) and in PF (Cases 29 DY and 27 FB), the percentage of CPNs was at least 5%. The percentage of CPNs was highly variable across cases. This variability could not be accounted for by the efficacy of the transport of the neural tracer, as there was no correlation (*r* = −0.239; *p* = 0.159) between the total number of labeled neurons and the percentage of CPNs considering all cases (Table 2). In spite of this variability, the percentage of CPNs tended to be higher for the premotor (frontal motor areas other than F1; mean = 14%, range 8–18%) and the prefrontal areas (mean = 17%, range 6–26%) and lower for parietal even excluding PF (mean = 9%, range 5–15%). A rostrocaudal gradient was observed for the prefrontal and the frontal opercular areas. Statistical analysis revealed that the percentage of CPNs observed following tracer injections in the parietal areas was significantly lower than that observed after the tracer injections in prefrontal (ratio = 0.46; *p* = 0.001) and premotor (ratio = 0.54; *p* = 0.032) areas. After the two tracer injections in F1 (Cases 13 TB and 62 FR) and in PF (Cases 29 DY and 27 FB), the percentage of CPNs was quite low even in Cases 62 FR and 29 DY in which the total number of labeled neurons was relatively high.

The homotopic callosal projection, expressed as the percentage of CPNs in the contralateral area corresponding to the injected one with respect to the total number of CPNs, in the majority of the cases was less than 50% and highly variable across cases (Fig. 2B). Specifically, after the tracer injections in the superior parietal lobule (SPL), frontal opercular, mesial premotor areas, and rostral area 12r, the percentage of homotopic CPNs was particularly low, ranging from 10% to 30%. No correlation (*r* = 0.007; *p* = 0.967) was found between the percentage values of all CPNs and those of homotopic CPNs. In the two cases of tracer injections in F1, the percentage of homotopic CPNs was markedly different. However, it is worth noting that total number of CPNs in Case 13 TB was very low with respect to Case 62 FR (Table 2). It is also possible that this variability in the callosal connectivity derives from differences in the location of the injection sites within F1.

Accordingly, these data show that the contribution of the callosal input to the connectivity pattern of prefrontal, premotor, and parietal areas can be quite robust and that for most of these areas the contribution of the heterotopic callosal projections prevails on that of the homotopic ones.

**Table 2:** Number and percentages of labeled neurons in the ipsilateral and contralateral hemisphere and in the homotopic area

Case	TOTAL number (ipsi + contra)	Number IPSI	Number CONTRA	% CONTRA	Number HOMOT.	% HOMOT.
64FB 46d (r)	15,919	11,804	4115	25.8%	2374	57.7%
60FB 46d (i)	27,710	24,904	2806	10.1%	1380	49.2%
58FB 46d (c)	21,139	18,676	2463	11.7%	1272	51.6%
48FB 12r (r)	15,157	11,244	3913	25.8%	427	10.9%
48DY 12r (r)	12,629	10,327	2302	18.2%	460	20.0%
52 FB 46v (i)	15,240	13,429	1811	11.9%	667	36.8%
52 DY 46v (i)	12,600	10,582	2018	16.0%	1594	79.0%
59FB 12r (i)	15,504	12,311	3193	20.6%	2076	65.0%
30FB 45B	25,591	23,951	1640	6.4%	799	48.7%
56FB GrFO	27,857	22,604	5253	18.9%	1502	28.6%
56DY PrCO	11,028	10,256	772	7.0%	162	21.0%
42FB DO	19,703	18,765	938	4.8%	257	27.4%
30DY F5a	5729	5289	440	7.7%	337	76.6%
42DY F5a	5346	4670	676	12.6%	302	44.7%
30CTBg F5p	23,019	20,993	2026	8.8%	551	27.2%
18FB F4	22,695	18,715	3980	17.5%	1255	31.5%
12DY F2	16,781	14,702	2079	12.4%	1767	85.0%
11FB F2	13,166	11,040	2126	16.1%	1075	50.6%
13FB F7	23,338	19,440	3898	16.7%	1864	47.8%
11DY F7	15,211	12,462	2749	18.1%	1376	50.1%
65FB F3	7353	6291	1062	14.4%	249	23.4%
65DY F6	18,596	16,003	2593	13.9%	556	21.4%
13 TB F1	2079	2030	49	2.4%	3	6.1%
62FR F1	33,812	33,566	246	0.7%	156	63.4%
27DY Opt	19,080	17,939	1141	6.0%	436	38.2%
27 TB Opt	7755	7128	627	8.1%	358	57.1%
29 TB PG	8580	7456	1124	13.1%	895	79.6%
29FB PFG	17,073	16,281	792	4.6%	271	34.2%
29DY PF	31,273	30,900	373	1.2%	175	46.9%
27FB PF	12,758	12,645	113	0.9%	69	61.1%
74FB AIP	6726	6106	620	9.2%	266	42.9%
74DY AIP	16,366	13,900	2466	15.1%	639	25.9%
73DY MIP	64,758	61,135	3623	5.6%	974	26.9%
72FB MIP	23,839	21,927	1912	8.0%	432	22.6%
72DY PEip	66,017	62,312	3705	5.6%	1094	29.5%
73FB PEip	23,528	20,556	2972	12.6%	458	15.4%

The percentage of neurons in the contralateral hemisphere is calculated relative to the total number of labeled neurons (ipsi + contra) and the percentage of CPNs in the homotopic area relative to the total number of CPNs

### Heterotopic callosal connectivity

In general, the majority of labeled heterotopic CPNs was located in areas adjacent to the homotopic one and their areal distribution partially reproduced the ipsilateral connectivity pattern.

**Fig. 3:**
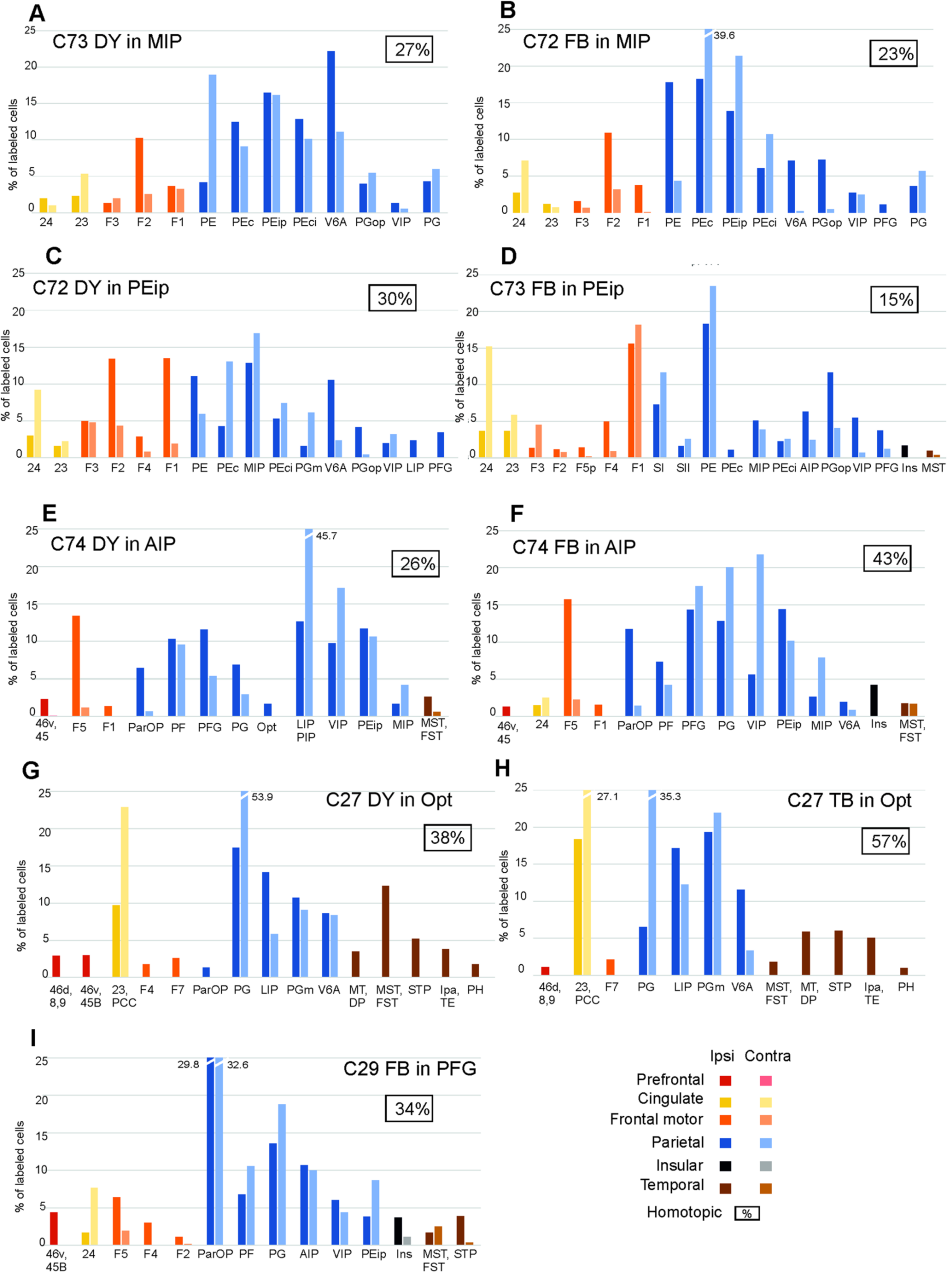
Percentage areal distribution of the labeled neurons observed after tracer injections in parietal areas in the ipsilateral (ipsi = 100%, darker colors) and the contralateral hemisphere (contra = 100%, lighter colors) outside the injected and the homotopic area. The percentage of labeled CPNs observed in the homotopic area with respect to the total amount of CPNs is indicated in the top right part of each panel; only cases in which homotopic projections were < 70% are shown, whereas the other cases are shown in Supplementary Fig. 1. Each graph shows only areas in which the ipsilateral labeling was > 1%. AIP, anterior intraparietal area; FST, fundal superior temporal area; Ins, insula; LIP, lateral intraparietal area; MIP, medial intraparietal area; MST, middle temporal superior area; MT middle temporal area; Opt, occipito-parieto temporal area; ParOp, parietal operculum; PCC, posterior cingulate cortex; STP, superior temporal polysensory area; VIP, ventral intraparietal area

**Fig. 4:**
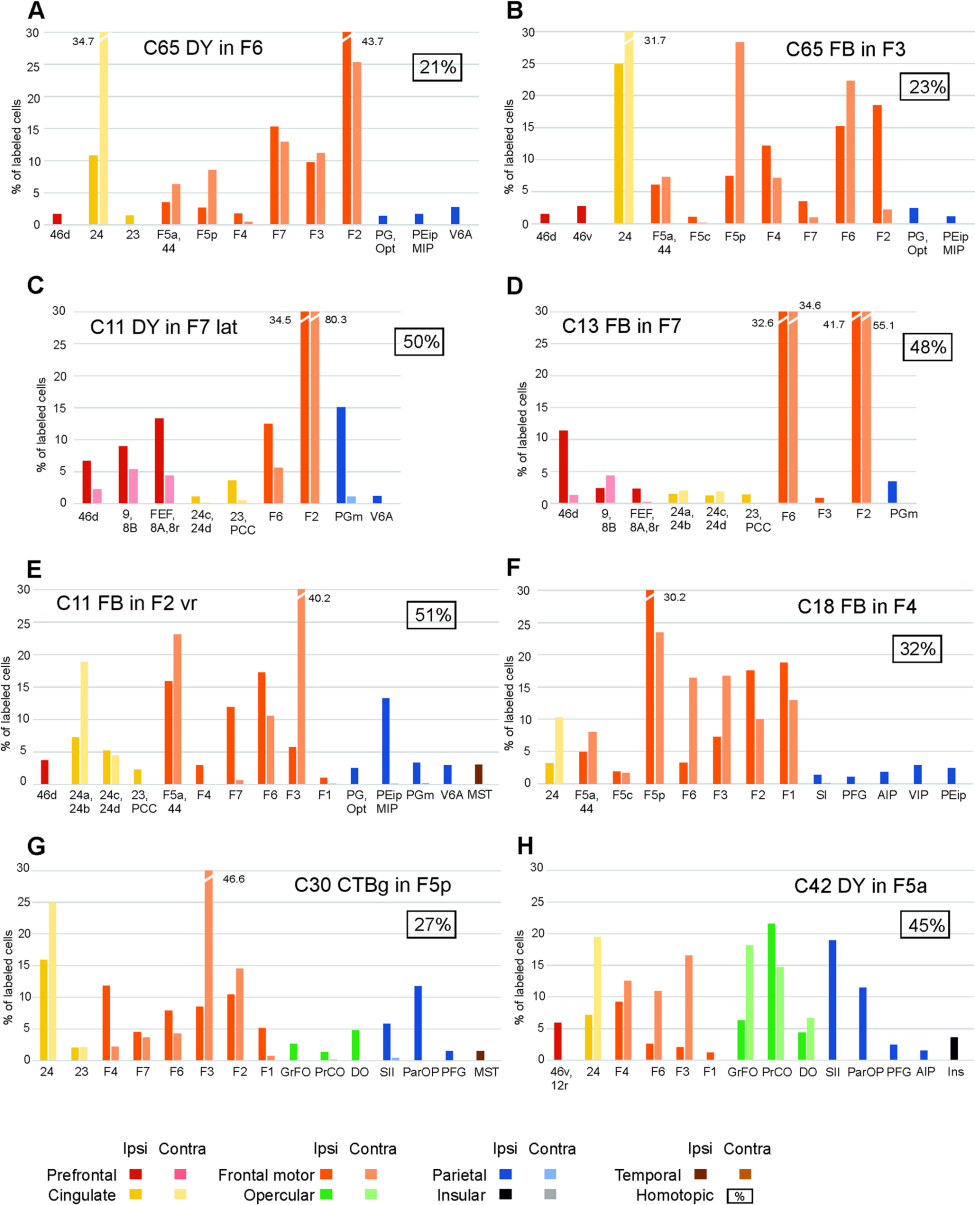
Percentage areal distribution of the labeled neurons observed after tracer injections in premotor areas in the ipsilateral and the contralateral hemisphere outside the injected and the homotopic area. FEF, frontal eye fields. Conventions and other abbreviations as in Fig. 3

**Fig. 5:**
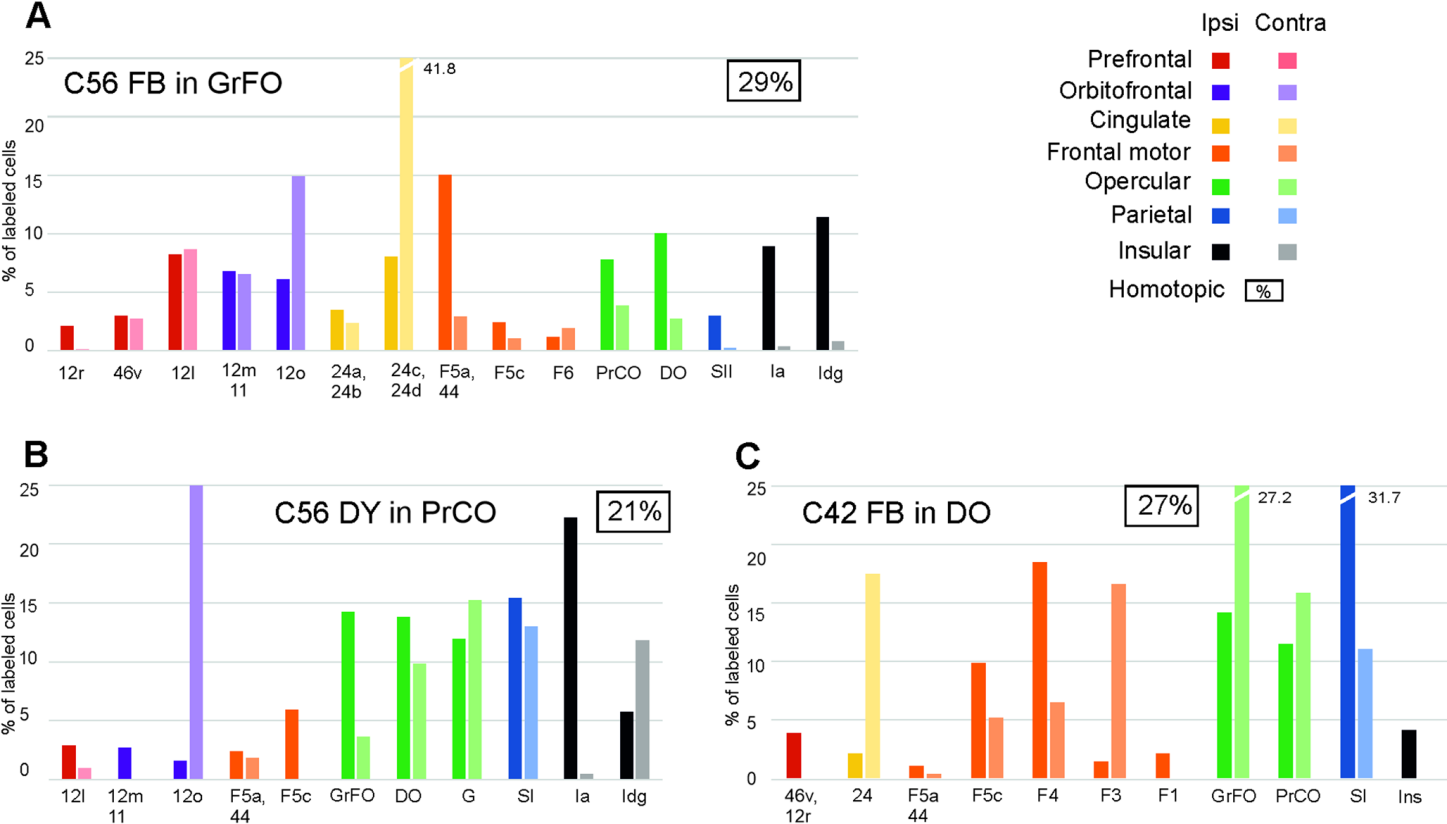
Percentage areal distribution of the labeled neurons observed after tracer injections in opercular areas in the ipsilateral and the contralateral hemisphere outside the injected and the homotopic area. DO, dorsal opercular; GrFO, granular frontal opercular; Ia, agranular insula; Idg, disgranular insula; PrCO, precentral opercular. Conventions and other and abbreviations as in Figs. 3 and 4

**Fig. 6:**
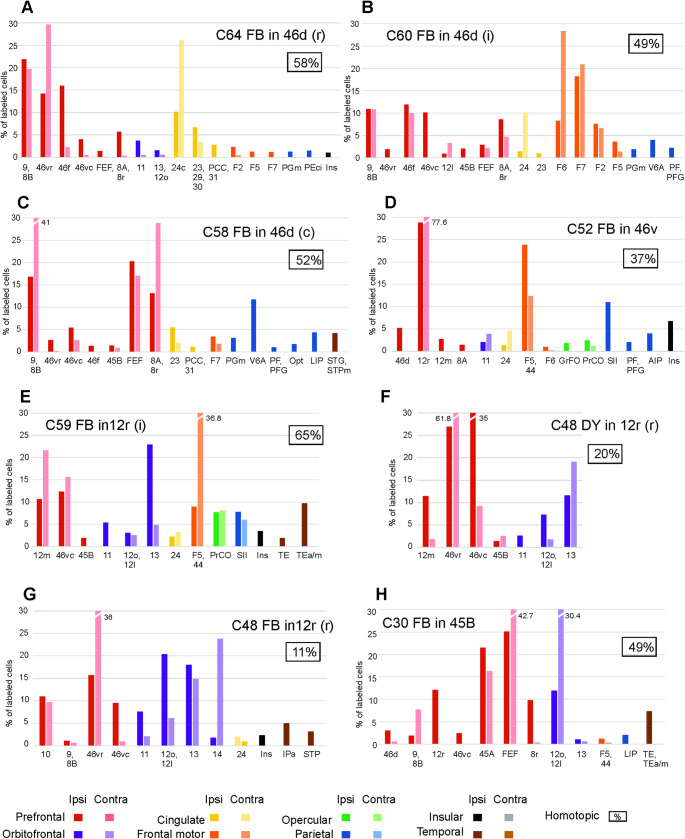
Percentage areal distribution of the labeled neurons observed after tracer injections in prefrontal areas in the ipsilateral and the contralateral hemisphere outside the injected and the homotopic area. Conventions and abbreviations as in previous figures

After the tracer injections in the SPL, heterotopic callosal labelling involved mostly other SPL areas, but also *distant* premotor, cingulate, and inferior parietal lobule (IPL) areas, thus quite well reproducing the ipsilateral connectivity pattern (Fig. 3A–D). Heterotopic callosal projections to IPL areas involved mostly other IPL areas (Fig. 3E–I; Supplementary Fig. 1, C, D and H). *Distant* callosal projections originated from SPL and cingulate areas, but not from premotor and prefrontal areas. After the tracer injections in premotor areas, there were relatively rich heterotopic projections originating from almost all the other premotor areas and the cingulate cortex (Fig. 4; Supplementary Fig. 1G). Contralateral parietal areas were virtually devoid of labeled cells. Other callosal projections included those from frontal opercular areas to F5a and from prefrontal areas to F7. After the tracer injection in area F5a (Case 30 DY) there were heterotopic projections only from F6 and 24, but also from ventrolateral prefrontal areas 46v and 12r (Supplementary Fig. 1 F). In the two cases of tracer injections in F1, sparse heterotopic CPNs were located in caudal premotor areas F3, F2, F4 and in area 24 (Supplementary Fig. 1 A, B). The distribution of the heterotopic projections to frontal opercular areas involved almost all the areas labeled in the ipsilateral hemisphere (Fig. 5). Finally, heterotopic callosal projections to prefrontal areas originated from other even *distant* prefrontal areas, from orbitofrontal, frontal opercular, premotor and cingulate areas (Fig. 6; Supplementary Fig. 1E). In most cases, parietal, temporal, and insular areas were devoid of labeling.

### Laterality index of the areal input to the studied areas

**Fig. 7:**
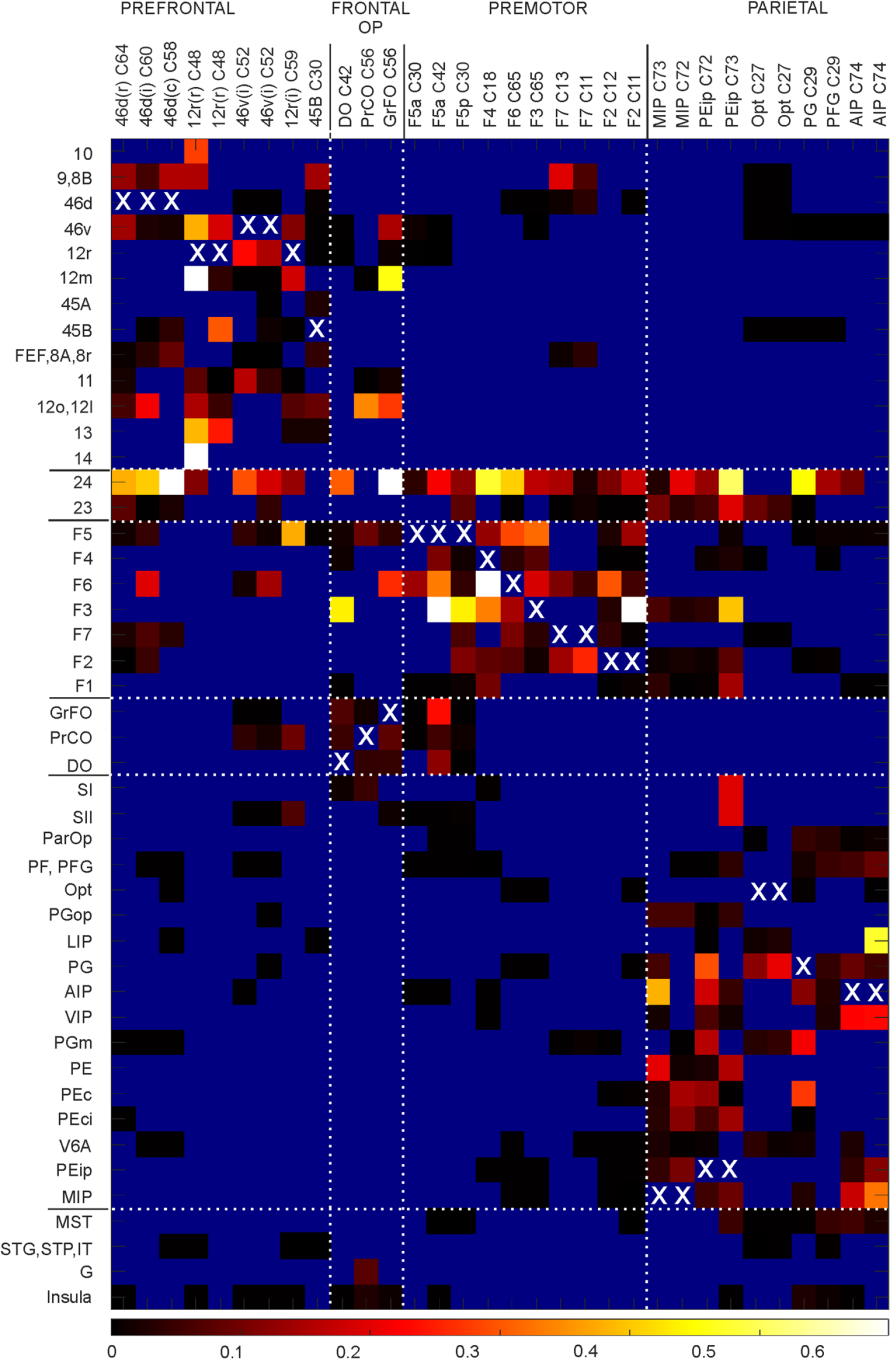
Heatmap of laterality index (number of labeled CPNs/ipsilateral labeled neurons in a given area) calculated for those areas in which the ipsilateral labeling was > 1%. White X mark corresponding areas. For area 14 in Case 48 12r (r) the laterality index was 4.8

As shown above, there were differences in the percentage distribution of labeled neurons in the ipsilateral vs. contralateral hemisphere. To see which areas tend to have a higher proportion of callosal projections, we calculated for each area in which the ipsilateral labeling was > 1% the ratio between the number of contralateral and ipsilateral labeled neurons. The results of this analysis (Fig. 7) showed that area 24 and several premotor areas tend to have more extensive callosal projections, that is they were often labeled bilaterally. Furthermore, in several cases the laterality index of the projections from areas 24, F6, F3 and some orbitofrontal areas was > 0.3, that is the number of labeled neurons in the contralateral area was at least about one third of the number of labeled neurons in the ipsilateral area.

### Laminar distribution of CPNs

The laminar distribution of the CPNs labeled after tracer injections in the various areas under study largely varied according to the projecting and/or the target area. Accordingly, any given area could be a target of differentially distributed CPNs in the various input areas and, in turn, could show different laminar distribution patterns of CPNs according to the target area. Furthermore, the observed laminar distribution patterns of the CPNs not necessarily were the same of those observed in the corresponding area of the ipsilateral hemisphere.

**Fig. 8:**
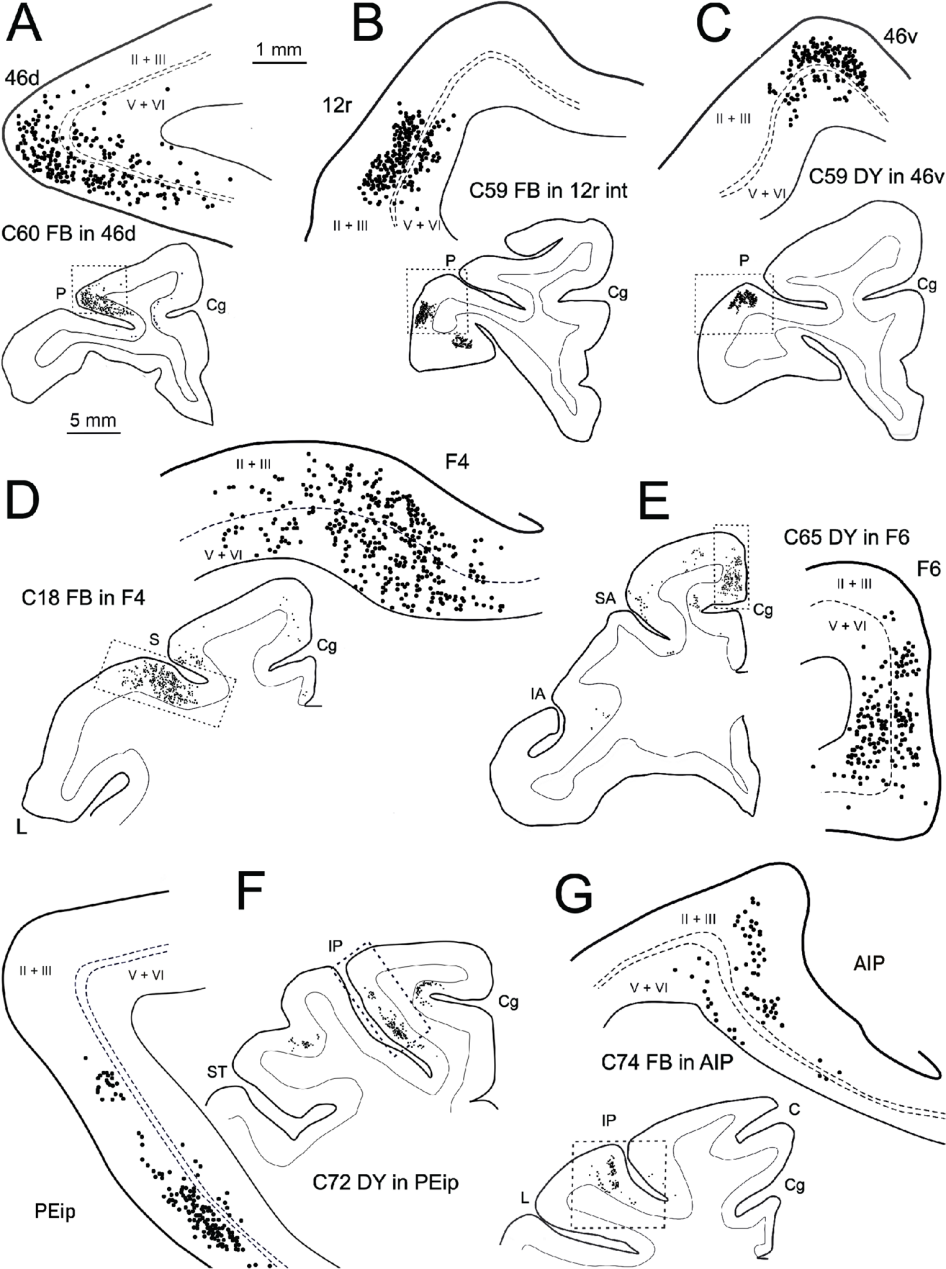
Drawings of representative sections showing the laminar distribution of CPNs in the homotopic area after tracer injections in prefrontal (**A**–**C**), premotor (**D**, **E**), and parietal (**F**, **G**) areas. Dashed lines in **A**–**C**, **F**, and **G** mark the location of layer IV, whereas in D and E the location of the border between layer III and V. Cg, cingulate sulcus; other abbreviations as in Fig. 1

**Fig. 9:**
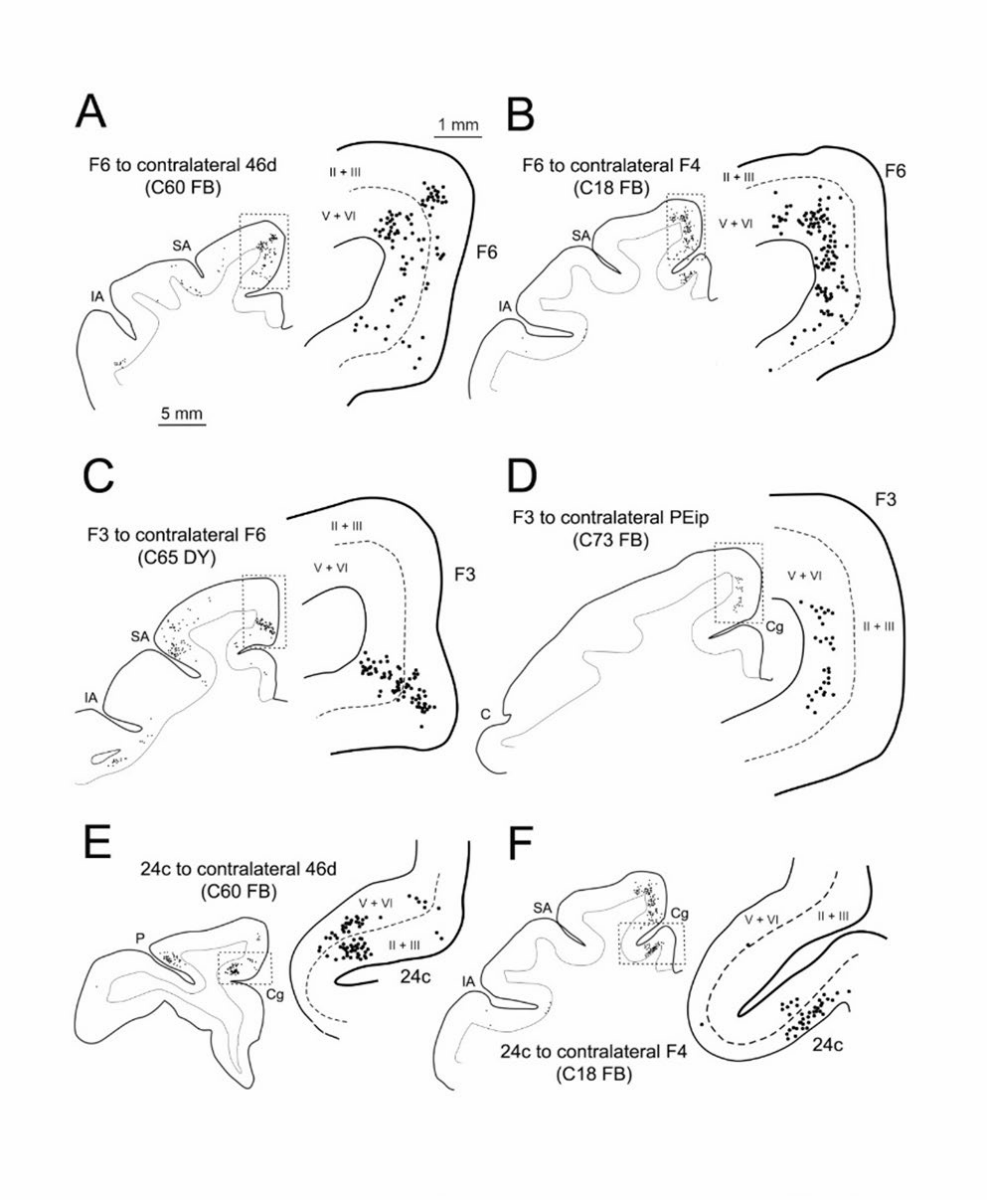
Drawings of representative sections showing the laminar distribution of heterotopic CPNs in areas F6 (**A**, **B**), F3 (**C**, **D**), and 24c (**E**, **F**). Dashed lines in all panels mark location of the border between layer III and V. Abbreviations as in Figs. 1 and 8

Homotopic callosal projections to different sectors of area 46d originated predominantly from layer III (Fig. 8A), thus showing a *feedforward* pattern. A similar laminar distribution pattern of CPNs was observed for the prefrontal heterotopic projections from areas 46v and 9. In contrast, extraprefrontal heterotopic projections to intermediate 46d from area F6, F7, and 24 showed a bilaminar distribution of the CPNs (Fig. 9A, E; *lateral* pattern). Caudal prefrontal heterotopic projections to caudal 46d showed an almost balanced distribution of CPNs in the superficial and deep layer (*lateral* pattern). In the ipsilateral areas 46v and 24 labeled neurons showed a *lateral* and a *feedforward* pattern, respectively.

As for area 46d, homotopic callosal projections to ventrolateral prefrontal areas 45B, 46v, and 12r showed a *feedforward* pattern (Fig. 8B, C). The same pattern was observed for the heterotopic callosal projections originating from adjacent or close prefrontal areas (FEF and 12l for area 45B; 12r for 46v; 46v and 12m for 12r), for those from the orbitofrontal cortex, and for those from F5 to intermediate area 12r. In contrast, ipsilateral projections to 46v and 12r displayed in general a *lateral* pattern, except for those originating from orbitofrontal and cingulate areas, which showed a *feedback* pattern.

All the various premotor areas were targets of homotopic projections and of heterotopic projections originating from other premotor areas showing a bilaminar distribution of CPNs (*lateral* pattern Figs. 8D and E and 9C), except for the projections from contralateral F6 to F2d and ventral premotor (Fig. 9B), those from area 24c/d to all premotor areas (Fig. 9F), and those from area 9/8B to F7, which showed a *feedback* pattern. These laminar connection patterns were similar to those observed in the ipsilateral hemisphere.

The opercular frontal areas GrFO, PrCO, and DO were targets of homotopic and heterotopic callosal projections from adjacent or close areas (PrCO, DO, F5, 12l, and 12o for GrFO; GrFO, DO, F5, and 12o for PrCO; GrFO and PrCO for DO) showing a *feed-forward* pattern, whereas those from area 12m/11 to GrFO and those from area 24 to DO showed a *feedback* pattern. In contrast, the projections from the corresponding areas of the ipsilateral hemisphere showed a *lateral* distribution pattern of labeled neurons.

In the parietal cortex, the homotopic callosal projections to all the various SPL and IPL studied areas showed a *feedforward* pattern (Fig. 8F, G). A similar pattern was also observed in all the major heterotopic projections from parietal areas, whereas those from frontal motor and cingulate areas to MIP (F1, F2, F3, 24c/d) and PEip (F1, F3, 24c/d) showed a *feedback* pattern (Fig. 9D). This pattern was the same observed in the corresponding areas of the ipsilateral hemisphere. Heterotopic callosal projections from area 23 to MIP and PEip showed a *feedforward* and a *feedback* pattern, respectively. In the ipsilateral parietal areas the labeled cells showed a *lateral* distribution.

Altogether, the present data, summarized in Table 3, suggest that homotopic callosal projections and heterotopic ones originating from adjacent or close areas of the same cortical sector tend to show a *feedforward* pattern for prefrontal, frontal opercular, and parietal areas and a *lateral* pattern for frontal motor areas. *Distant* heterotopic projections, i.e. those originating from areas located in other cortical regions very often show a *feedback* pattern. This was the case for the projections from contralateral area 13 to rostral 12r, from contralateral F5 to intermediate 12r, from contralateral F6 to F2, F4, and F5a, from contralateral frontal motor areas to PEip and MIP, and from contralateral area 24 to F3, F6, F4, F5, PrCO, DO, PEip, and MIP.

**Table 3:** Laminar distribution patterns of the homotopic (bold), the main intraregional (plain text), and extraregional heterotopic (italics) callosal connections of the areas under study

Area	Feedforward	Lateral	Feedback
46d	**46d**, 46v*, 9*	FEF*, 8A-8r* , *F6*,* F7*, 24*	
46v	**46v**, 12r*, F5*		
12r	**12r**, 46v*, 12m* , 12o* , 13*, F5*		
45B	**45B**, FEF*, 12l* *		
GrFO	**GrFO**, PrCO*, DO*, F5*, 12l*, 12r*		*12m/11 *
PrCO	**PrCO**, GrFO*, DO*, F5*, 12r*		
DO	**DO**, GrFO*, PrCO*		*24*
F5		**F5**, F4, F3, F2	F6*, 24*
F4		**F4**, F5, F2, F3, F1	F6, 24*
F2		**F2**, F3, F5	F6*, 24*
F7		**F7**, F6, F2	9/8B*
F3		**F3**, F6, F4, F5	24*
F6		**F6**, F7, F3, F2 F5	24*
F1		**F1**	
PF	**PF**		
PFG	**PFG**, ParOp*, PF*, PG*, AIP*, VIP*, PEip*		
PG	**PG**, ParOp*, PFG*, Opt*, PEc*, MIP*, V6A*		
Opt	**Opt**, PG*, LIP*, PGm, V6A		
AIP	**AIP**, PF*, PFG*, PG*, VIP*, PEip*, MIP*,		
MIP	**MIP**, PEip*, PE*, PEc*, Peci*, V6A*, PG*		*F1*,* F2*,* F3, 24c/d*,
PEip	**PEip**, MIP*, PE*, PEc*, PEci*, V6A, * VIP*, PGop*,		*F1*,* F3*,* 24c/d*,* 23*

*Laminar distribution pattern of labeled cells different from that inthe corresponding ipsilateral area

## Discussion

The present study is the first in providing quantitative information on the contribution of the callosal afferences to the overall cortical input to different prefrontal, frontal motor, frontal opercular, and parietal areas. Despite some variability across cases, the results showed that the callosal projections can represent an important component (up to 25%) of the overall cortical afferences to a given area and that in most cases these projections originate from non-corresponding areas (heterotopic callosal projections). Furthermore, the results showed that in several cases the areal distribution of CPNs was more restricted with respect to that of the labeled cells in the ipsilateral hemisphere. However, there were areas in which the distribution of CPNs was more symmetrical and their proportion compared with that of the ipsilateral projecting neurons was relatively high. Finally, the laminar origin of callosal projections could differ from that of the ipsilateral ones and varied according to the projecting and/or the target area.

### Callosal connectivity in the non-human primates

Early descriptions of the callosal connectivity in the macaque brain have been performed using silver impregnation techniques after partial or complete section of the corpus callosum or ablation of different cortical sectors (e.g. Ebner and Myers 1962; Myers 1962; Jones and Powell 1969; Pandya and Vignolo 1969, 1971; Pandya et al. 1969; Karol and Pandya 1971; Pandya et al. 1971). Altogether, these studies showed that the callosal connectivity involves most cortical areas except the anterior part of the temporal lobe, most of area V1, and sensorimotor fields where the hand is represented.

Several studies based on neural tracers injections have then described in detail the distribution of the callosal connections of prefrontal (Schwartz and Goldmann-Rakic, 1984; Barbas and Pandya 1984), frontal motor (Gould et al. 1986; Rouiller et al. 1994; Liu et al. 2002; Marconi et al. 2003; Boussaoud et al. 2005; Lanz et al. 2017; Dancause et al. 2007; Jenny 1979; Muakkassa and Strick 1979; Leichnetz 1986; Jones et al. 1979), somatosensory (Killackey et al. 1983; Manzoni et al. 1984, 1986), posterior parietal (Caminiti and Sbriccoli 1985; Seltzer and Pandya 1983; Andersen et al. 1985; Neal 1990), temporal (Cipolloni and Pandya 1985), and extrastriate visual (Van Essen et al. 1982) areas. The areal distribution of the CPNs observed in the present study is in substantial agreement with that shown in those studies in which neural tracers have been injected in the same areas. Specifically, the scarcity of callosal connectivity observed after the tracer injections in F1 (Cases 13 TB and 62 FR) could be accounted for by the involvement by the injection site of the hand field of this area, which in many studies was found to have very poor, if any, callosal connections. It was originally claimed that the hand area of M1 lacks callosal connections (Brodal 1992; Pandya and Vignolo 1971; Jones et al. 1979). Other studies, based on relatively large tracer injections involving the hand field, have described callosal connections of M1 (Jenny 1979; Leichnetz 1986; Muakkassa and Strick 1979). The present study is in line with other studies (Gould et al. 1986; Rouiller et al. 1994) showing that the hand representation of M1 has indeed callosal connections, but scarce as compared to other cortical areas. A scarcity of callosal connections was observed also in the two cases of injections in PF (Cases 29 DY and 27 FB). To our knowledge, there is only one study (Neal 1990) describing qualitatively the callosal connections of PF, which appeared as in our study quite limited.

In all the above mentioned studies, no quantitative data have been provided on the weight of the callosal with respect to the total afferences to the studied areas. Furthermore, data on the percentage distribution of CPNs have been provided only in studies focused on the callosal connectivity of frontal motor areas (Rouiller et al. 1994; Liu et al. 2002; Marconi et al. 2003; Boussaoud et al. 2005; Lanz et al. 2017). In agreement with the present data, these studies have highlighted a relatively high variability in the areal distribution of CPNs across different cases, which can be at the basis of the differences across studies including the present one. These differences were particularly evident comparing the percentage of homotopic CPNs. Specifically, after neural tracer injections in ventral premotor areas the percentage of homotopic CPNs ranged from about 20 to 80% and after neural tracer injections in dorsal premotor areas from about 20 to 50% (Marconi et al. 2003; Boussaoud et al. 2005; Lanz et al. 2017), which is comparable to the data of the present study. After neural tracer injections in medial premotor areas, the percentage of homotopic CPNs varied from 32 to 65% (Rouiller et al. 1994; Liu et al. 2002), whereas in the present cases it was about 20–25%. In spite of this large variability, that can be also accounted for by the dimension and the location of the various injection sites, all these studies including the present one are in substantial agreement in showing that the homotopic area tends to be the most labeled contralateral area (with some exceptions, see also ventral and dorsal caudal premotor injections in Boussaoud et al. 2005), but also that the various premotor areas have a complex pattern of heterotopic connectivity with the contralateral premotor areas, especially with the medial premotor and area 24. Furthermore, area F7 is a target of heterotopic projections from prefrontal areas. Lanz et al. (2017) have observed also heterotopic afferences from prefrontal to the ventral premotor cortex, originating mostly from area PrCO, which in our subdivision is considered as an opercular frontal area.

Furthermore, a quantitative estimate of the proportion of homotopic vs. heterotopic projections in the mouse, marmoset, and human brain has been obtained using diffusion-weighted imaging (Szczupak et al. 2023). In all the three species, heterotopic projections were estimated to be more than 70% of the total callosal connectivity with differences across cortical regions. Though this value appears to be higher than that observed in our data, this study is in line with the notion that heterotopic connections heavily contribute to the callosal connectivity.

Finally, the present data suggest that the laminar origin of callosal projections largely varies according to both their areal origin and termination, which explains the marked differences in this aspect of the callosal connectivity observed in several previous studies. Specifically, as observed by Schwartz and Goldmann-Rakic (1984) we found that homotopic and intraprefrontal heterotopic projections to prefrontal areas originate from superficial layers. However, we found that extraprefrontal callosal projections tend to show a bilaminar origin. In agreement with data of Liu et al. (2002), Rouiller et al. (1994) and Johnson et al. (1989) we found that premotor areas are targets of homotopic and heterotopic intrapremotor projections showing in most cases a bilaminar origin, but in some cases, including heterotopic projections from area 24, a *feedback* pattern. In agreement with Andersen et al. (1985) and Johnson et al. (1989), we found that homotopic and intraparietal heterotopic projections to parietal areas originate mostly from superficial layers. However, heterotopic projections from frontal and cingulate motor areas originated mostly from deep layers.

### Interhemispheric connectivity

The corpus callosum is a distinctive feature of placentals brain. It is by far the largest fiber tract in the brain interconnecting corresponding and non-corresponding areas of the two cerebral hemispheres through about 56 million axons in the rhesus monkey (LaMantia and Rakic 1990) and about 200 million axons in humans (Aboitiz et al. 1992). It is largely agreed (see Innocenti 1986; Aboitiz and Montiel 2003) that callosal connections between sensorimotor areas are responsible for the process of midline fusion, that is the integration of sensorimotor processing carried out in the two hemispheres for a unitary perception and bimanual coordination. Other studies, for example, have highlighted a role of the callosal connectivity of the motor areas in contralateral learning transfer, that is the transfer of motor skills to one hand after motor practice of the contralateral one and have correlated microstructural changes in the corpus callosum with long-term bimanual motor training (Pauwels and Gooijers 2023; Nuara et al. 2025). Furthermore, callosal connections between higher order areas could be important for integration and synchronization of neural activity in the cortical networks of the two hemispheres. Recently, according to Innocenti et al. (2022), the callosal connections “could drive their post-synaptic targets when acting with other inputs, thus playing a conditional driving or modulatory role, which depends on task contingencies”.

The vast majority of CPNs establish excitatory glutamatergic synapses, though in some cases axons terminate on inhibitory neurons (Innocenti 1986; Conti and Manzoni 1994). In rodents, a relatively high proportion of CPNs send projections to either contralateral and ipsilateral areas, or to contralateral areas and striatum (see e.g., Fame et al. 2011; Pal et al. 2024). CPNs projecting to both contralateral areas and striatum in macaques were observed by Parent and Parent (2006). However, studies in macaques based on double labeling experiments showed that CPNs and ipsilateral association neurons in prefrontal, frontal motor, and parietal areas are mostly distinct cell populations (GoldmannRakic and Schwartz 1982; Andersen et al. 1985; Johnson et al. 1989). Furthermore, in macaque, layer III CPNs and layer III cortico-cortical ipsilateral neurons in the dorsolateral prefrontal cortex markedly differ in their gene expression (Arion et al. 2023). 

The present qualitative and quantitative analysis of the callosal connections highlights some aspects of the complex pattern of interhemispheric connectivity in the macaque brain. First, our data show that heterotopic connections heavily contribute to the callosal connectivity, though with variability across different cortical regions. Specifically, based on the distribution of CPNs observed in a relatively high number of tracer injections in prefrontal, frontal opercular, frontal motor, and parietal areas we found that some areas, such as area 24 and medial premotor areas tend to project to almost similar sets of ipsilateral and contralateral areas. These same areas showed in general a relatively high laterality index, that is a relatively high proportion of CPNs compared to the neurons projecting ipsilaterally to a specific area. Furthermore, area 24 and medial premotor areas were among those areas showing a higher proportion of crossed corticostriatal projections, whereas parietal areas, which tend to have a more restricted callosal corticocortical connectivity, lack crossed corticostriatal projections (Borra et al. 2022). Tractographic data in humans showed that medial premotor areas are those frontal motor areas showing the densest and more extensive callosal connectivity (Fling et al. 2013; Ruddy et al. 2017). Altogether, these data suggest regional differences and common organizational principles in the interhemispheric corticocortical and corticostriatal connectivity.

The extensive callosal cortico-cortical and corticostriatal connectivity of the two medial premotor areas F3 and F6 could provide the neural substrate for the proposed role of these areas in some general aspects of motor control. Specifically, F6, also referred to as pre-supplementary motor area (pre-SMA) is considered to play a supramotor role in motor control (Nachev et al. 2008), being involved in planning and/or inhibiting goal-directed actions (Tanji and Shima 1996), error monitoring (Fu et al. 2023), procedural learning of motor sequences (Hikosaka et al. 1999), and in in switching from automatic to volitionally controlled action in rhesus macaque monkeys (Isoda and Hikosaka 2007). Furthermore, F3, also referred to as SMA-proper, is involved in postural control and, in particular, in postural adjustments preceding voluntary movements (Massion 1992; Jacob et al. 2009) and in bimanual coordination (Brinkman 1984). In macaques, after lesions of the primary motor cortex, there is a functional reorganization bilaterally in premotor areas (Moreau-Debord et al. 2021) and sprouting of callosal projections to the ipsilesional premotor cortex can mediate an almost full recovery of motor performance (Hamadjida et al. 2012). These data suggest that callosal connectivity can play an important role in recovery of motor functions together with reorganization of descending motor pathways (Darling et al. 2011). In humans, plastic changes in the interhemispheric heterotopic connectivity of the SMA with premotor areas is believed to be responsible for the recovery from the SMA syndrome, that is a temporary loss of motor function observed after surgical lesions in correspondence of the medial premotor cortex (Vassal et al. 2017; Pinson et al. 2022). Similarly, the extensive callosal cortico-cortical and corticostriatal connectivity of the rostral cingulate cortex could provide the substrate for its modulatory role in emotional processing, cognitive functions, and autonomic system responses. In humans, bilateral cingulotomy can be effective for treatment of refractory pain (Sharim and Pouratian 2016; Mc Benedict et al. 2024). 

Second, the present data show asymmetries in the interhemispheric parietofrontal connectivity. Specifically, frontal motor areas virtually lacked heterotopic callosal projections from parietal areas, whereas parietal areas showed *feedback* heterotopic callosal projections from frontal motor areas. It is well-known that parietofrontal connections are crucial for sensorimotor transformations in which sensory processing leads to the activation of motor programs in the premotor cortex. In this context, the present data suggest that information flow from parietal to frontal motor areas appears to be strictly ipsilateral, whereas signals related to motor programs can be broadcasted bilateraterally in the opposite direction.

In conclusion, the present data, along with previous data on the crossed corticostriatal connectivity, provide a general framework, though still not complete, of the interhemispheric connectivity in the primate brain. Specifically, they could be useful for a better understanding of the interactions between the two hemispheres in sensorimotor integration, and cognitive functions (e.g., Innocenti et al. 2022; Pauwels and Gooijers 2023). Furthermore, they could serve as reference for interpreting and understanding the neuronal mechanisms underlying the role of cortical areas in the functional recovery after lesions in the contralateral hemisphere (e.g., Carmichael et al. 2017; Grefkes and Fink 2020).

## Supplementary Information

Below is the link to the electronic supplementary material.

Supplementary Material 1

## Data Availability

The data that support the findings of this study are available from the corresponding author upon reasonable request.
