# Supplementary material for: Qualitative and quantitative analysis of the callosal projections to prefrontal, frontal motor, and parietal areas in the macaque monkey
Source: Brain Struct Funct. 2025 Dec 17;231(1):3. doi: 10.1007/s00429-025-03060-x (PMC12712016; doi:10.1007/s00429-025-03060-x)
Supplement: Supplementary file 1 — Supplementary Material 1 [file 429_2025_3060_MOESM1_ESM.docx]

**Supplementary Figure 1**


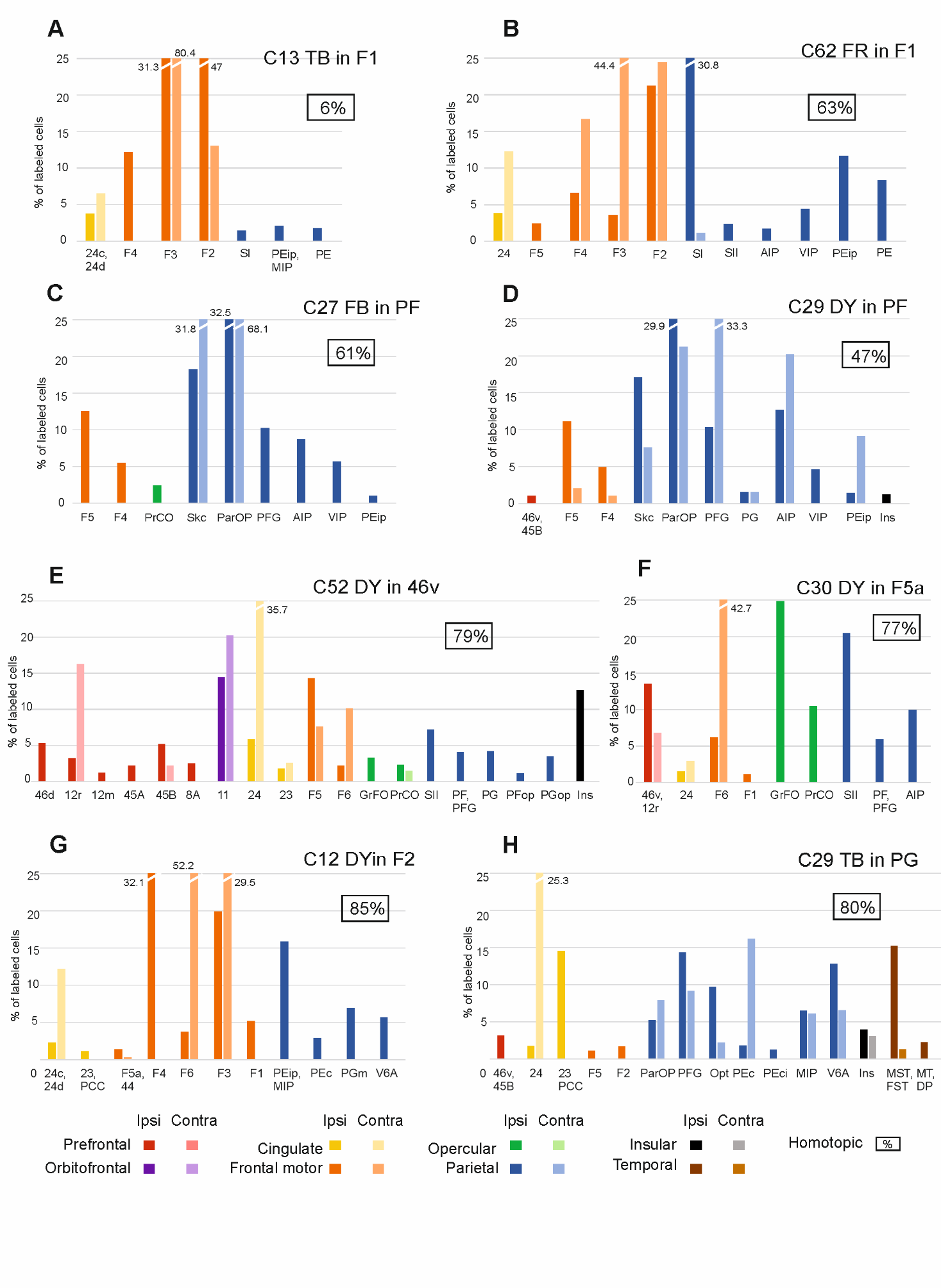


**Supplementary Figure 1**: Percentage areal distribution of the labeled neurons observed in the ipsilateral (ipsi=100%, darker colors) and the contralateral hemisphere (contra =100%, lighter colors) outside the injected and the homotopic area in those cases in which the percentage of CPNs was less than 5%, or homotopic CPNs were >70%. Each graph shows only areas in which the ipsilateral labeling was >1%. The percentage of labeled CPNs observed in the homotopic area with respect to the total amount of CPNs is indicated in the top right part of each panel. AIP, anterior intraparietal area; FST, fundal superior temporal area; GrFO, granular frontal opercular; Ins, insula; LIP, lateral intraparietal area; MIP, medial intraparietal area; MST, middle temporal superior area; MT middle temporal area; Opt, occipito-parieto temporal area; ParOp, parietal operculum; PCC, posterior cingulate cortex; PrCO, precentral opercular; Skc, somatic koniocortex; STP, superior temporal polysensory area; VIP, ventral intraparietal area.
